# Protease inhibitors enhance extracellular collagen fibril deposition in human mesenchymal stem cells

**DOI:** 10.1186/s13287-015-0191-1

**Published:** 2015-10-15

**Authors:** Sejin Han, Yuk Yin Li, Barbara Pui Chan

**Affiliations:** Tissue Engineering Laboratory, Department of Mechanical Engineering, The University of Hong Kong, Room 711, Haking Wong Building, Pokfulam Road, Hong Kong Special Administrative Region, China

**Keywords:** Protease inhibitor, Collagen fibril deposition, Matrix remodeling, Mesenchymal stem cells, Collagen, Intracellular, Extracellular

## Abstract

**Introduction:**

Collagen is a widely used naturally occurring biomaterial for scaffolding, whereas mesenchymal stem cells (MSCs) represent a promising cell source in tissue engineering and regenerative medicine. It is generally known that cells are able to remodel their environment by simultaneous degradation of the scaffolds and deposition of newly synthesized extracellular matrix. Nevertheless, the interactions between MSCs and collagen biomaterials are poorly known, and the strategies enhancing the extracellular matrix deposition are yet to be defined. In this study, we aim to investigate the fate of collagen when it is in contact with MSCs and hypothesize that protease inhibition will enhance their extracellular deposition of collagen fibrils.

**Methods:**

Specifically, human MSCs (hMSCs) were exposed to fluorescence-labeled collagen with and without intracellular or extracellular protease inhibitors (or both) before tracing the collagen at both intracellular and extracellular spaces.

**Results:**

Collagen were internalized by hMSCs and degraded intracellularly in lysosomes. In the presence of protease inhibitors, both intracellular collagen fibril growth and extracellular deposition of collagen fibrils were enhanced. Moreover, protease inhibitors work synergistically with ascorbic acid, a well-known matrix deposition-enhancing reagent, in further enhancing collagen fibril deposition at the extracellular space.

**Conclusion:**

These findings provide a better understanding of the interactions between hMSCs and collagen biomaterials and suggest a method to manipulate matrix remodeling and deposition of hMSCs, contributing to better scaffolding for tissue engineering and regenerative medicine.

**Electronic supplementary material:**

The online version of this article (doi:10.1186/s13287-015-0191-1) contains supplementary material, which is available to authorized users.

## Introduction

Collagen represents one of the major components of extracellular matrix (ECM) in mammalian tissues. It has been widely used as a naturally occurring biomaterial in tissue engineering and regenerative medicine because of its excellent biocompatibility, negligible immunogenicity, specific interaction with growth factors and cell adhesion molecules, biodegradability, and the fact that it permits cell ingrowth and matrix remodeling [1]. Collagen is a biomacromolecule with triple-helical peptide. When present at a concentration higher than the threshold (e.g., 25 μg/ml) with appropriate temperature, pH, and ionic strength, collagen molecules would self-assemble to form collagen meshwork, mimicking that in the ECM of the native tissues. We previously developed a scaffolding technology platform, namely collagen microencapsulation [2], which entraps living cells such as mesenchymal stem cells (MSCs) [2], chondrocytes [3], and nucleus pulposus cells [4].

MSCs represent promising cell sources for tissue engineering and regenerative medicine because of their multiple differentiation potential, self-renewal capability, easy accessibility, and hypo-immunogenic nature [5–8]. Previous studies demonstrated that MSCs adhered, proliferated, and differentiated on collagen via integrins and ERK activation [9–11]. Our own work showed that human MSCs (hMSCs) are able to express integrins α2β1, α5β1, and αvβ3 in the collagen matrix environment [12].

The ECM niche is a complex network of topographical, mechanical, and biochemical factors regulating cellular-fate processes, including cell proliferation, differentiation, shape, and migration [13–16]. Matrix metalloproteinases (MMPs) that cleave the protein components of the ECM as well as non-ECM molecules play a central role in matrix remodeling [17]. Several studies demonstrated that MMPs are involved in migration, proliferation, and differentiation of MSCs [18–20]. Moreover, cysteine proteases are involved in ECM remodeling as demonstrated in bone resorption, wound healing, and arterial remodeling [21]. In this study, we hypothesize that inhibiting intracellular and extracellular proteases will affect the matrix remodeling, including collagen fibril deposition of MSCs. Specifically, we aim to study how collagen matrix material interacts with MSCs, including collagen internalization and degradation as well as the effect of intracellular and extracellular protease inhibitors on collagen fibril deposition. This study provides a better understanding of the interactions between cells and collagen matrix and suggests a method to promote ECM meshwork deposition of hMSCs, contributing to rationalized scaffolding for tissue engineering and regenerative medicine.

## Methods

### 2D monolayer culture of human mesenchymal stem cells

hMSCs from bone marrow were kindly provided by G.C.F. Chan, of the Department of Paediatrics and Adolescent Medicine, the University of Hong Kong, and cultured as monolayers as previously described [22]. All procedures were approved by the Combined Clinical Ethics Committee of the University of Hong Kong and Hong Kong West Cluster Hospitals of Hospital Authority. In brief, hMSCs were cultured in growth medium—Dulbecco’s modified Eagle’s medium-low glucose (DMEM-LG), 10 % fetal bovine serum, 100 U/ml penicillin, 100 mg/ml strepto-mycin and 2 mM L-glutamine—at 37 °C in a humidified atmosphere with 5 % CO_2_. The growth medium was replaced every 3–4 days. At around 80 % confluence, hMSCs were trysinized with trypsin-EDTA (0.05 %) before re-suspending in full medium for subsequent experiments. Cells at P6 were used for subsequent experiments.

### Collagen internalization and degradation in hMSC monolayer culture

To study whether hMSCs in monolayer cultures would internalize the exogenously supplemented collagen matrix, rat tail type I collagen (BD Biosciences, Bedford, MA, USA) was fluorescently labeled before exposure to hMSC cultures, as previously described [23]. In brief, type I collagen solution in 0.02 N acetic acid was re-suspended in 2 mM HCl after salting out with 0.9 M NaCl. Then pH of the type I collagen solution in 2 mM HCl was raised by sodium carbonate buffer (0.5 M NaCl and 0.1 M sodium carbonate) to pH of 7.5 to approximately 8.3. Alexa Fluor 488 or Alexa Fluor 647 carboxylic acid succinimidyl ester (Invitrogen, part of Thermo Fisher Scientific, Waltham, MA, USA) was transferred to collagen solution and incubated at room temperature for 30 min. Labeling reaction was stopped by lowering pH, and the labeled collagen was purified by salt precipitation and dialysis against 0.02 N acetic acid extensively. The concentration of the fluorescence-labeled collagen was then determined by a Circular Dichroism machine at 221 nm (lambda spectrum; PerkinElmer, Waltham, MA, USA). Serum-free culture medium (DMEM-LG medium with 1 % penicillin-streptomycin and 1 % L-glutamine) containing the fluorescently labeled collagen at a concentration of 25 μg/ml was freshly prepared at 4 °C. hMSCs were then incubated with the collagen-containing serum-free medium for up to 24 h before inspection for internalized collagen under confocal laser scanning fluorescence microscopy (LSM710; Carl Zeiss, Oberkochen, Germany). In separate batches of experiments, DQ FITC-labeled collagen (1 %) (Life Technologies, Carlsbad, CA, USA), which fluoresces if degraded, was used to incubate with hMSC monolayer cultures for 24 h to study whether the internalized collagen was degraded by the cells.

### 3D microencapsulation of hMSCs in type I collagen

To study whether extracellular collagen matrix can be internalized by hMSCs when the cells are exposed to the matrix in three-dimensional (3D) configuration, hMSCs were microencapsulated in collagen as previously reported [2]. hMSCs were mixed with 1 % FITC-labeled bovine type I collagen solution (Life Technologies) in an ice bath with a cell seeding density of 1 × 10^5^ or 5 × 10^5^ cells/ml and a collagen concentration of 1 or 2 mg/ml. Tiny droplets of the mixtures (2.5 μl) were pipetted into petri dishes, the bottoms of which were covered with parafilm to prevent adhesion of the constructs to the substratum. The collagen-hMSC mixtures were gelated when incubated at 37 °C in a humidified atmosphere with 5 % CO_2_ for 45 min. The gelated droplets were then free-floated in growth medium to allow contraction before harvesting at different time points (3, 19, and 23 h) for inspection of the internalized collagen.

### Inhibition of collagen degradation using protease inhibitors and enhancing collagen synthesis by ascorbic acid in hMSCs

Glass coverslips (10 mm in diameter) were placed into a 4- or 24-well plate and sterilized with 70 % ethanol and ultraviolet irradiation. hMSCs were then seeded on the glass coverslips at a density of 1 × 10^4^ per well and cultured for overnight. The cells were washed with phosphate-buffered saline (PBS) and serum-free medium. After incubation for 1 h in serum-free medium, medium was replaced with serum-free medium containing fluorescently labeled type I collagen at a concentration of 10 μg/ml. Cells were moved to 37 °C in a humidified atmosphere with 5 % CO_2_ for the desired time periods (4, 24, 48, and 72 h). To study the effects of inhibiting matrix degradation on ECM deposition, the free collagen present in the serum-free medium was labeled with Alexa Fluor 488 such that when matrix is deposited, fluorescence fibrils would be observed. To inhibit collagen degradation, an intracellular matrix degradation inhibitor, namely E64D (20 mM), or a broad-spectrum ECM degradation inhibitor, namely GM6001 (25 mM) was previously optimized, or the combination of both inhibitors was supplemented to hMSCs in serum-free medium. To enhance collagen synthesis by hMSCs, 0.5 mM of ascorbic acid (Sigma-Aldrich, St. Louis, MO, USA) was supplemented in culture medium either in serum-free medium or in medium with serum. To visualize intracellular collagen, extracellular collagen and collagen fibrils were removed by bacterial collagenase (50 units/ml) for 10 min. At appropriate time points, incubation was terminated by rinsing in PBS and fixing with 4 % paraformaldehyde for 10 min at room temperature before subsequent immunofluorescence evaluation.

### Fluorescence or immunofluorescence evaluation

Exogenously supplemented collagen was fluorescence-labeled either by Alexa 488 or similar fluorescence probes as previously demonstrated [23] or by DQ-FITC labeled collagen, which becomes fluorescent when being degraded. Newly synthesized type I collagen by hMSCs was evaluated by immunofluoresence. Specifically, samples were fixed with 4 % PBS-buffered paraformaldehyde for 10 min at room temperature. Fixed samples were permeabilized by 0.02 % Triton X-100 (Sigma-Aldrich) for 10 min at room temperature in the dark. Endogenous peroxidase activity was blocked with H_2_O_2_. Samples were blocked with 2 % normal horse serum for non-specific binding and then incubated with a primary antibody against human type I collagen (rat anti-human type I collagen antibody in 1:200 dilution; Chondrex, Redmond, WA, USA) overnight at 4 °C. Samples were further incubated with Alexa Fluor 488- or 647-conjugated secondary antibody in the dark for 1 h at room temperature. Samples were washed with PBST (PBS with 0.05 % Tween20) for 5 min four times and then mounted with anti-fading fluorescent mounting medium with 4ʹ,6-diamidino-2-phenylindole (DAPI) (Electron Microscopy Sciences, Hatfield, PA, USA). Some subcellular organelles were labeled to provide the reference for intracellular location. To visualize lysosomes, which are the organelles that internalize collagen, 50 nM LysoTracker (Molecular Probe, 1:1000) was added to the culture medium for 60 min at 37 °C prior to fixation with 4 % paraformaldehyde. To visualize cell membrane, CellMask Red (Life Technologies, 2.5 mg/ml) was added to the culture medium for 8 min after the lysotracker labeling. DAPI was used to label the nuclei.

### Quantitative analysis of internalized collagen and extracellularly deposited collagen fibrils

To analyze the amount of collagen internalized in the intracellular space or deposited in the extracellular space quantitatively, hMSCs were incubated with culture medium containing fluorescently labeled collagen monomers for various period of time. Quantitative analysis of internalized Alexa 488-labeled collagen was determined by measuring the total fluorescence, using a microplate reader (Safire II; Tecan, San Jose, CA, USA), in the cell lysate fraction collected up to 50 h after discarding the extracellular fraction containing deposited collagen fibrils by digestion with excess bacterial collagenase (Sigma-Aldrich). In separate experiments, after discarding the culture medium, the collagen fibrils deposited in the extracellular space were digested by collagenase, and the amount of collagen was determined by measuring the total fluorescence in this fraction.

### Data presentation and statistics

Quantitative data on the percentage of collagen content were presented as mean and standard error of the mean. The normality assumption was verified with the Kolmogorov-Smirnov test and the equal variance assumption was verified by Levene’s test to justify the use of parametric tests. The collagen content among different treatment groups at different time points was compared by using two-way analysis of variance (ANOVA) with appropriate *post hoc* tests. For data with equal variance assumed, Bonferroni’s test was used. For data without equal variances, Dunnett’s T3 test was used. SPSS 19.0 (IBM Corporation, Armonk, NY, USA) was used to execute all analyses, and the statistical significance was set at 0.05.

## Results

### Collagen was internalized and degraded by hMSCs in both 2D and 3D models

**Fig. 1:**
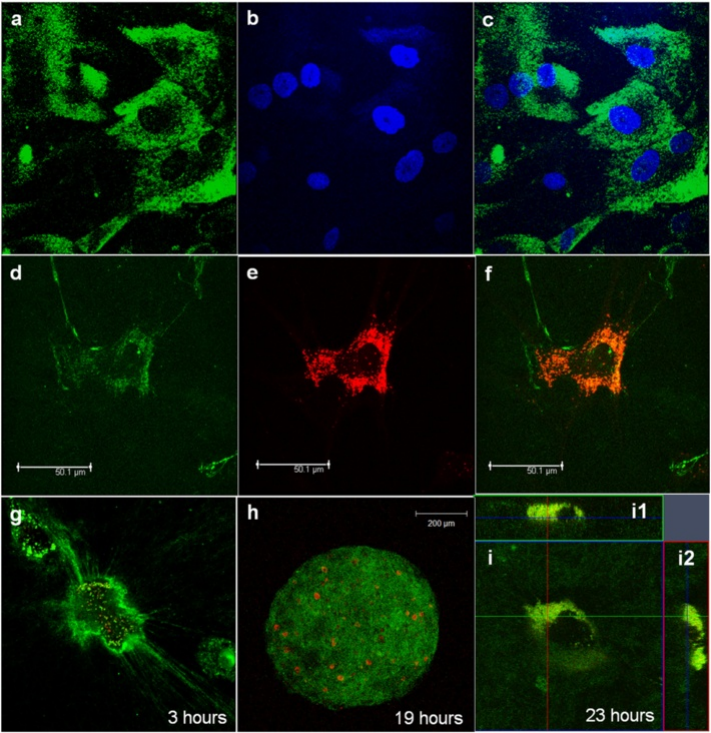
Collagen internalization and degradation by hMSCs (P6). **a**-**f** Two-dimensional monolayer culture: **a**-**c** 1-hour incubation in serum-free medium (*green*: Alexa Fluor 488 labeled collagen; 25 μg/ml; *blue*: nucleus labeled by DAPI); **d**-**f** 24-hour incubation in serum-free medium where DQ FITC-labeled collagen was 10 % of total collagen content (20 μg/ml) (*green*: fluorescein-conjugated DQ-collagen type I; *red*: lysosomes). **g**-**i** Three-dimensional culture in collagen microspheres at different time points: **g** 2 mg/ml type I rat collagen with cell density at 1 × 10^5^ per ml at 3 h after encapsulation; **h** 1 mg/ml type I rat collagen with cell density at 5 × 10^5^ per ml at 19 h after encapsulation; **i** 1 mg/ml type I rat collagen with cell density at 5 × 10^5^ per ml at 23 h after encapsulation; **i1** and **i2** Side views of hMSCs inside a collagen microsphere (*green*: 1 % fluorescein-labeled bovine collagen; *red*: LysoTracker-labeled cell). *DAPI* 4ʹ,6-diamidino-2-phenylindole, *FITC* fluorescein isothiocyanate, *hMSC* human mesenchymal stem cell

Figure 1a-c showed the presence of fluorescence (Alexa 488)-labeled collagen inside the human MSCs as soon as 1 h after incubation with serum-free culture medium containing collagen. The internalized collagen was degraded as shown by the presence of fluorescence staining of fluorescein-conjugated DQ-collagen (Fig. 1d), which becomes fluorescent only when collagen is being degraded. Moreover, the degrading collagen also co-localized (Fig. 1f) with lysosomes (Fig. 1e), which are the subcellular organelles responsible for internalizing molecules, at 24 h after incubation. In a 3D collagen microencapsulation model, internalization of collagen was also demonstrated (Fig. 1g-i). Specifically, Fig. 1g showed that internalization of fluorescence-labeled collagen was noted as early as 3 h after encapsulation. Figure 1h showed the image projection of an MSC-collagen microsphere in which hMSCs were labeled with lysosomes and randomly distributed in fluorescence-labeled collagen meshwork, at 19 h after encapsulation. Figure 1i showed a magnified view of a single hMSC in the 3D collagen microsphere at 23 h after encapsulation where the yellowish co-localization of the lysosome and the fluorescence-labeled collagen was obvious. Figure 1i1 and i2 showed the side views of the cell where the fluorescence-labeled collagen co-localized with lysosomes in the cytoplasm within the same cell, demonstrating intracellular internalization.

### Protease inhibitors enhanced the deposition of collagen fibrils at the extracellular space

**Fig. 2:**
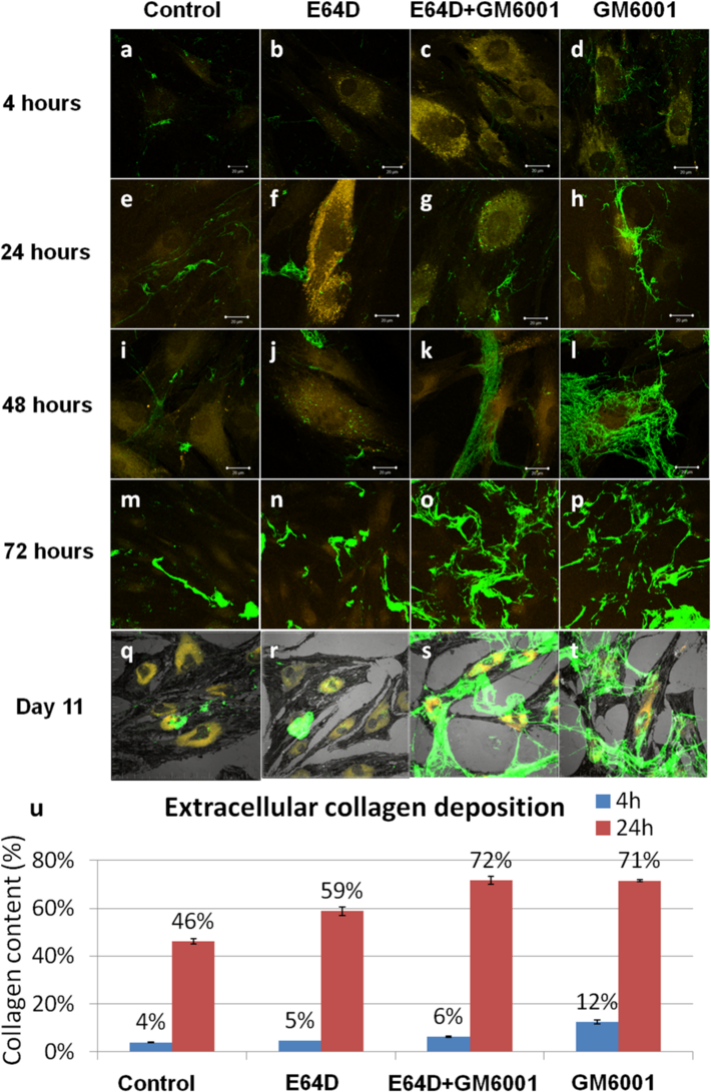
Extracellular collagen fibril formation by hMSCs in different treatment groups at different time points. **a**-**d** 4 h. **e**-**h** 24 h. **i**-**l** 48 h. **m**-**p** 72 h. **q**-**t** Day 11. **a**, **e**, **i**, **m**, **q** Control (normal medium). **b**, **f**, **j**, **n**, **r** Inhibition of intracellular matrix degradation by intracellular cysteine proteinase inhibitor E64D. **c**, **g**, **k**, **o**, **s** Inhibition of extracellular matrix degradation by wide range collagenase inhibitor GM6001. **d**, **h**, **l**, **p**, **t** Inhibition of both intracellular and extracellular matrix degradation. E64D (20 μM); GM6001 (25 μM); *green*: Alexa Fluor 488-labeled collagen type I at 10 μg/ml; *orange*: Lysosomes. **u** Quantitative analysis of percentage of collagen content in extracellular fibril fraction under different treatments and at different time points (n = 2 with duplicates). *hMSC* human mesenchymal stem cell

In addition to internalizing and degrading the exogenously supplemented collagen monomers, hMSCs organize and form thick and long collagen fibrils at extracellular space, as shown by the green fluorescent collagen fibrils, as soon as 4 h after incubation (Fig. 2a), and it was more obvious at 24 h (Fig. 2e) in the control group. Thicker and longer collagen fibrils were continuously formed at 48 and 72 h after incubation (Fig. 2i, m). With presence of protease inhibitors (Fig. 2b-d, f-h, j-l, n-p), there is an obvious trend of enhanced collagen fibril formation at the extracellular space. Specifically, in the presence of intracellular protease inhibitor, there was a slight increase in the extracellular collagen fibril formation (Fig. 2b, f, j, n) as compared with the control group. With the presence of extracellular collagenase inhibitor, extensive deposition of collagen meshwork at the extracellular space was noted (Fig. 2c, d, g, h, k, l, o, p) and was particularly obvious at 48 (Fig. 2k, l) and 72 (Fig. 2o, p) hours after incubation. On day 11, extensive accumulation of thick collagen bundles at the extracellular space was noted in the presence of extracellular protease inhibitor (Fig. 2s, t). In separate experiments, quantitatively analyzing the amount of collagen deposited at the extracellular space after collagenase digestion at 4 and 24 h after exposure of hMSCs to exogenously supplemented collagen monomers was shown in Fig. 2u. There was an overall increasing trend of extracellularly deposited collagen fibrils. At 4 h, 12 % of collagen was deposited as fibrils in the extracellular protease inhibitor (GM6001) group, but only less than 6 % of extracellularly deposited collagen was found in other groups, including the control and the groups with intracellular protease inhibitor (ED64). At 24 h, collagen fibril deposition was significantly increased in all groups that those treated with extracellular protease inhibitors (GM6001 alone and a combination of both ED64 and GM6001) showed more than 70 % collagen in the deposited fibril fraction. On the other hand, the group treated with intracellular protease inhibitor (ED64) showed approximately 60 % collagen in the deposited fibril fraction as compared with less than 50 % in the control group. Two-way ANOVA showed that both the treatment group (*P* < 0.001) and the time factor (*P* < 0.001) significantly affected collagen deposition. Bonferroni’s *post hoc* tests showed that all groups were significantly different from one another (*P* ≤ 0.003).

### Extracellular protease inhibitors promoted intracellular collagen fibril formation and growth without changing the total collagen internalized

**Fig. 3:**
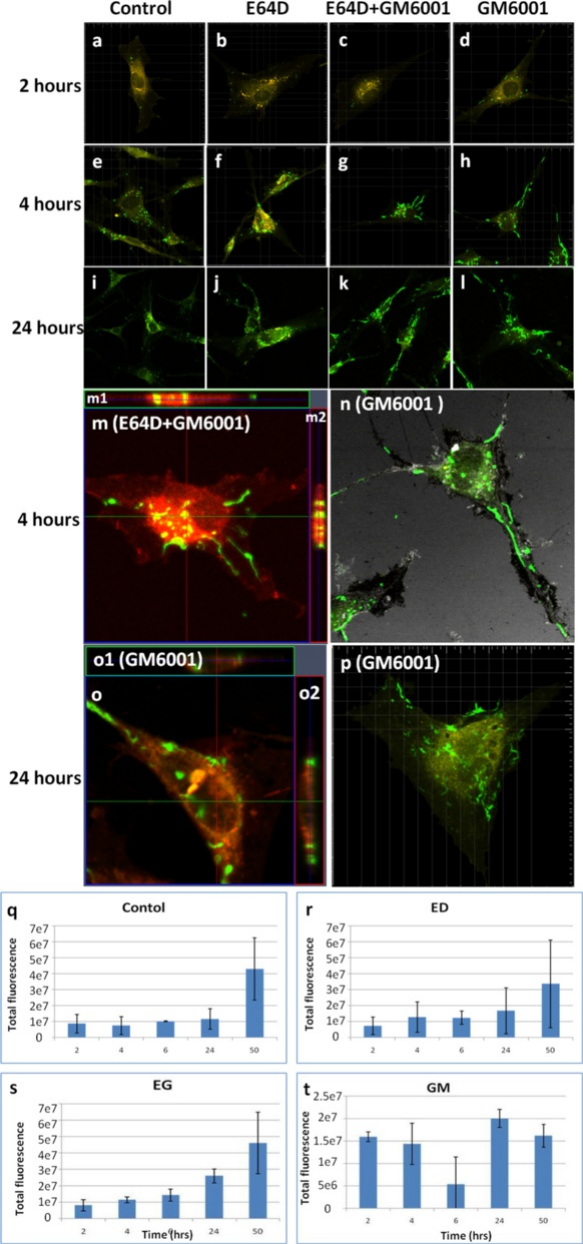
Intracellular collagen fibril formation and growth by hMSCs in different groups at different time points after removal of extracellular collagen fibrils by treatment with bacterial collagenase type VI. **a**-**p** Visualization of internalized collagen after collagenase treatment. **a**-**d** 2 h. **e**-**h**, **m**, **n** 4 h. **i**-**l** 6 h. **o**, **p** 24 h. **a**, **e**, **i** Control. **b**, **f**, **j** Inhibition of intracellular matrix degradation by cysteinase inhibitor (E64D). **c**, **g**, **k**, **m** Combination of both intracellular (E64D) and extracellular (GM6001) protease inhibitors. **d**, **h**, **l**, **n**-**p** Inhibition of extracellular matrix degradation by broad-spectrum protease inhibitor (GM6001). *m 1*–*2, o1-2* Side views of hMSCs showing that the location of the stained fibrils was within the intracellular space. **q**-**t** Quantitative measurement of total fluorescence intensity of internalized collagen per cell on images at 63× (mean + standard deviation, n = 2 to 8). **q** Control. **r** Inhibition of intracellular matrix degradation by cysteine proteinase inhibitor (E64D). **s** Combination of both intracellular (E64D) and extracellular (GM6001) protease inhibitors. **t** Inhibition of extracellular matrix degradation by broad-spectrum protease inhibitor (GM6001). *Green*: Alexa Fluor 488-labeled collagen type I; *orange*: lysozyme; *red*: Plasma membrane; and *grey*: reflection. Collagen (10 mg/ml), E64D (20 μM), and GM6001 (25 μM) were used. *hMSC* human mesenchymal stem cell

To visualize the collagen fibril formation and growth in the intracellular space, hMSCs were continuously treated with collagenase, which removes the extracellularly deposited collagen. Figure 3 showed the presence of collagen fibrils in the intracellular space of hMSCs at different time points followed by collagenase treatment. There was not much collagen fibril at 2 h in most groups (Fig. 3a-c), but some short fibrils were noted in the group with extracellular protease inhibitor (Fig. 3d). At 4 h, more collagen was observed within the intracellular space in all groups (Fig. 3e-h), although long collagen fibrils were observed at the intracellular space only in groups treated with extracellular protease inhibitor (Fig. 3g, h), whereas in the group with intracellular protease inhibitor, short collagen fibrils were observed (Fig. 3f). More obvious accumulation of long collagen fibrils in groups with extracellular protease inhibitor was observed at 24 h (Fig. 3k, l). At high magnifications, the presence of elongated collagen fibrils within the intracellular space was found in groups treated with the extracellular protease inhibitor either in combination with the intracellular protease inhibitor (Fig. 3m) or alone (Fig. 3n-p). Moreover, internalized collagen co-localized with lysosomes in the same focal plane (Fig. 3m1 and 2, o1 and 2) at both 4 h (Fig. 3m, n) and 24 h (Fig. 3o, p). In separate experiments, quantitative analysis of collagen at the intracellular space was conducted via total fluorescence measurement in the cell lysates collected up to 50 h after discarding the extracellular collagen via collagenase digestion (Fig. 3q-t). It was noted that, in the group with extracellular protease inhibitor GM6001 alone, the quantity of collagen at the intracellular space did not show obvious change over time (Fig. 3t), even though collagen fibrils appeared longer. On the other hand, continuous increase in the total amount of internalized collagen from approximately 1 × 10^7^ to 7 × 10^7^ was noted in the control group (Fig. 3q) and the groups with intracellular protease inhibitor (Fig. 3r, s).

### Ascorbic acid and protease inhibitors work together in enhancing collagen fibril deposition

**Fig. 4:**
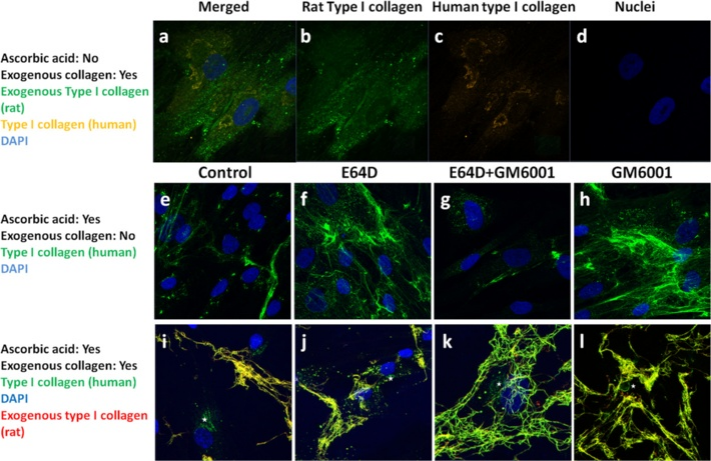
Collagen fibril deposition by hMSCs with and without exogenously supplemented collagen or ascorbic acid or both. **a**-**d** Immunofluorescence staining of exogenous type I collagen (rat) and newly synthesized type I collagen (human) in hMSCs without ascorbic acid but with exogenously supplemented collagen: **a** Merged channels. **b** Rat type I collagen. **c** Human type I collagen. **d** Nuclei. **e**-**h** Immunofluorescence staining of collagen type I in hMSCs with ascorbic acid but without exogenously supplemented collagen. **i**-**l** Immunofluorescence staining of human type I collagen and fluorescence-labeled exogenous rat type I collagen when vitamin C and exogenous collagen were co-supplemented. **e**, **i** Control. **f**, **j** Intracellular protease inhibitor E64D. **g**, **k** Combination of intracellular (E64D) and extracellular (GM6001) protease inhibitors. **h**, **l** Extracellular protease inhibitor GM6001. For (**e**-**l**), *green*: human type I collagen; *red*: Exogenously supplemented fluorescence-labeled rat tail collagen type I; *blue*: 4ʹ,6-diamidino-2-phenylindole (DAPI). *hMSC* human mesenchymal stem cell

In the absence of vitamin C, when removing the extracellularly deposited collagen fibrils by collagenase after a brief exposure to exogenous collagen, internalized rat collagen and newly synthesized human collagen were located intracellularly (Fig. 4a-c) while no collagen fibrils were deposited at the extracellular space (Fig. 4a-d). Vitamin C is known to stimulate collagen matrix deposition as revealed in Fig. 4e, where obvious collagen fibril deposition was noted even though no exogenous collagen was supplemented. Without exogenous supplementation of collagen, when intracellular protease inhibitor ED64 (Fig. 4f) or extracellular protease inhibitor GM6001 (Fig. 4h) was co-supplemented with vitamin C, more extensive collagen fiber meshwork was found, suggesting that vitamin C works synergistically with protease inhibitors in enhancing collagen fibril synthesis and deposition. Interestingly, there was little fibril deposition when both intracellular and extracellular protease inhibitors were used (Fig. 4g), suggesting that possible interactions between the intracellular and the extracellular protease inhibition pathways deserve further investigation. When exogenous collagen was supplemented to the culture medium, co-supplementation of either the intracellular or the extracellular protease inhibitor and vitamin C significantly increased the abundance of collagen fibrils deposited in the extracellular space in all groups (Fig. 4i-l), suggesting that vitamin C and protease inhibitors provide the optimal condition in enhancing ECM deposition in hMSCs. It is also noted that newly synthesized human collagen type I was found at the intracellular space (white stars) (Fig. 4i-l). It is also interesting to note that almost all collagen fibrils deposited at the extracellular space showed extensive co-localization with the exogenously supplemented fluorescence-labeled rat tail collagen and the newly synthesized human collagen type I (Fig. 4i-l), suggesting that the supplemented collagen was used as a template by hMSCs for matrix remodeling and deposition.

## Discussion

Here we report the interactions between collagen biomaterials and hMSCs and a method to promote extracellular collagen matrix deposition, therefore contributing to better scaffolding for hMSC-based tissue engineering and regenerative medicine. Upon exposure to exogenously supplemented collagen biomaterials, hMSCs internalized collagen and degraded them intracellularly. Three known pathways are involved in collagen turnover: (1) cleavage of collagen extracellularly by a group of secreted or membrane-associated MMPs [1], (2) cathepsin-mediated pathway [21], and (3) involvement of collagen fibrils binding to specific cell surface receptors followed by cellular internalization and lysosomal breakdown [24]. Previous studies have shown that phagocytosis of collagen is initiated by binding through α2β1 integrin receptors [25] or mediated by urokinase plasminogen activator receptor-associated protein (uPARAP/Endo180) [26] or both. hMSCs may involve the first and the third pathways to regulate collagen turnover. Our in-house studies showed that hMSCs expressed integrin α2β1 at the cell-collagen matrix interface (Additional file 1), which is known to mediate collagen internalization, as well as MMPs, including MMP1, the major collagenase degrading collagen matrix (Additional file 2), and MT1-MMP (Additional file 3), which is required to cleave collagen extracellularly before collagen can be internalized through the Endo180 pathway. Collagen internalization is a spontaneous and time-dependent response when cells such as fibroblasts are exposed to exogenous collagen. This phenomenon has been shown to be independent of MMPs and cysteine proteases in fibroblasts [27, 28]. Similarly, hMSCs are able to internalize collagen in all groups. The control group and those with intracellular protease inhibitors exhibited the time-dependent increase in the amount of internalized collagen, echoing the protease-independent collagen uptake and internalization [27, 28]. Interestingly, in the presence of the extracellular protease inhibitors, the amount of internalized collagen was maintained at a similar level throughout the incubation period, suggesting that the enhanced collagen fibril deposition at the extracellular space should be a result of enhanced inhibition of matrix degradation or increased export of collagen fibrils into the extracellular space.

Our working hypothesis is to inhibit protease-based collagen degradation so as to enhance the deposition of extracellular collagen matrix, which is an important step during matrix remodeling when hMSCs are in contact with collagen biomaterials. E64D is a membrane-permeable cysteine protease inhibitor, whereas GM6001 is a broad-spectrum extracellular inhibitor of MMPs. Indeed, we demonstrated that blockage of matrix proteases, particularly the extracellular ones, induced and enhanced collagen fibril deposition and accumulation, probably by (1) reducing matrix degradation or (2) enhancing intracellular and extracellular fibril formation and growth or both. Creemers et al. [29] demonstrated that collagen degradation was strongly reduced in periosteal tissue explants as a result of the inhibition of cysteine proteases or MMPs. Reduction of collagen degradation was accompanied by an accumulation of intracellular fibrillar collagen and decreased degradation of internalized collagen in fibroblasts treated with cysteine protease inhibitor [30]. Hao et al. [31] reported that GM6001 reduced collagen degradation by inhibiting both the activation of pro-MMPs and the activity of MMPs in keratocytes. We did demonstrate that increased intracellular and extracellular fibril formation and growth were observed, particularly in the GM6001 group, suggesting that this might be one of the mechanisms of the enhanced fibril deposition at the extracellular space. The presence of protease inhibitor but not the collagen internalization/degradation is crucial in facilitating the extracellular fibril deposition. This is because there was very little extracellular collagen fibril deposition when MSCs were exposed to exogenous collagen without protease inhibitors where we did see internalization and degradation (Fig. 4a-c) whereas extracellular collagen fibril deposition was seen only in the presence of protease inhibitors particularly the extracellular one (Fig. 4i-l).

Ascorbic acid is an essential cofactor for two enzymes involved in post-translational modifications of collagen [32] and increases collagen production [33, 34]. Fernandes et al. [35] demonstrated that the amount of collagen synthesized by hMSCs was decreased in the absence of ascorbic acid. This observation is in line with our data that little extracellular collagen fibril deposition was found in the absence of ascorbic acid but that fibril deposition was found in the presence of ascorbic acid with and without supplementation of exogenous collagen. It is evident that collagen degradation is stimulated whenever collagen expression and deposition are enhanced. For example, Shiga et al. [36] showed that ascorbic acid increased collagen type I expression but in the mean time induced the expression of collagenase-1 and hence collagen degradation. Ruangpanit et al. [37] reported that the fibrillar form of collagen induced MT1-MMP expression and MMP-2 activation in fibroblasts. In addition, Ishikawa et al. [38] demonstrated that vitamin C enhanced the expression of α2β1 integrin, which stimulated collagen internalization and degradation [25]. In the presence of protease inhibitor, particularly the extracellular one (GM6001), the collagen degradation induced by the presence of vitamin C is likely to be inhibited, thereby enhancing extracellular deposition of collagen fibrils. The extensive deposition of collagen fibrils is likely to be the result of the actions of both the vitamin C-mediated enhancement in collagen binding and post-translation of collagen as well as the protease inhibitor-mediated reduction in collagen degradation.

Our study highlights the complexity of matrix remodeling of hMSCs and the fact that manipulation of matrix proteases not only affects the matrix degradation but also influences matrix deposition. Whether this approach, manipulation of matrix degradation through treatment with different protease inhibitors, can be used to promote ECM deposition during MSC-based tissue engineering and regenerative medicine warrants further investigations, but we do have some evidence on the effects of collagen internalization, degradation, and protease inhibitor treatments on MSC self-renewal and differentiation potential. Specifically, internalization and degradation of collagen when MSCs are in contact with collagen are inevitable. It is likely that internalization and degradation do not significantly affect MSC self-renewal and differentiation. In our early study on the collagen encapsulation process [2], during which MSCs internalize collagen as shown in the present study (Fig. 1g-i), when MSCs were allowed to migrate out from the collagen matrix, their self-renewal capability and multiple differentiation potential were not affected at all [2]. Our lab has ongoing efforts in studying whether supplementation of protease inhibitors will affect differentiation of MSCs. Preliminarily, the addition of protease inhibitors does affect chondrogenic differentiation by shifting toward the formation of fibrocartilage rather than hyaline cartilage (private communication).

## Conclusions

The present study describes the interactions between human MSCs and exogenously supplemented collagen. This work demonstrates that ECM deposition can be enhanced by inhibition of protease matrix degradation. Upon exposure to collagen, hMSCs internalize and degrade collagen. In the presence of protease inhibitors, intracellular collagen fibril growth was noted while extracellular collagen fibril deposition was enhanced. The presence of ascorbic acid in addition to protease inhibitors further enhanced the deposition and accumulation of collagen fibrils at the extracellular space. This study provides better understanding on the interactions between hMSCs and collagen biomaterials and suggests a method to manipulate matrix remodeling and collagen deposition of hMSCs, contributing to better scaffolding for tissue engineering and regenerative medicine.

## Additional files

Additional file 1:
**Expression of integrin α2β1.** Immunopositive expression of integrin α2β1 for hMSC microencapsulated in collagen matrix. *hMSC* human mesenchymal stem cell. (TIFF 452 kb) Additional file 2:
**Expression and secretion of MMP1.** Enhanced MMP1 expression and secretion of hMSC upon encapsulation in collagen matrix. a Real-time PCR on MMP1 gene expression. b MMP1 secretion upon ELISA measurement. *ELISA* enzyme-linked immunosorbent assay, *hMSC* human mesenchymal stem cell, *MMP* matrix metalloproteinase, *PCR* polymerase chain reaction. (TIFF 121 kb) Additional file 3:
**Expression of MT1-MMP.** Immunopositive expression of MT1-MMP in hMSC microencapsulated in collagen matrix. *hMSC* human mesenchymal stem cell, *MMP* matrix metalloproteinase. (TIFF 496 kb)

## Abbreviations

3D: Three-dimensional
ANOVA: Analysis of variance
DAPI: 4ʹ,6-diamidino-2-phenylindole
DMEM-LG: Dulbecco’s modified Eagle’s medium-low glucose
ECM: Extracellular matrix, hMSC, Human mesenchymal stem cell
MMP: Matrix metalloproteinase
MSC: Mesenchymal stem cell
PBS: Phosphate-buffered saline
