# Supplementary figures and images for: Protease inhibitors enhance extracellular collagen fibril deposition in human mesenchymal stem cells
Source: Stem Cell Res Ther. 2015 Oct 15;6:197. doi: 10.1186/s13287-015-0191-1 (PMC4606504; doi:10.1186/s13287-015-0191-1)

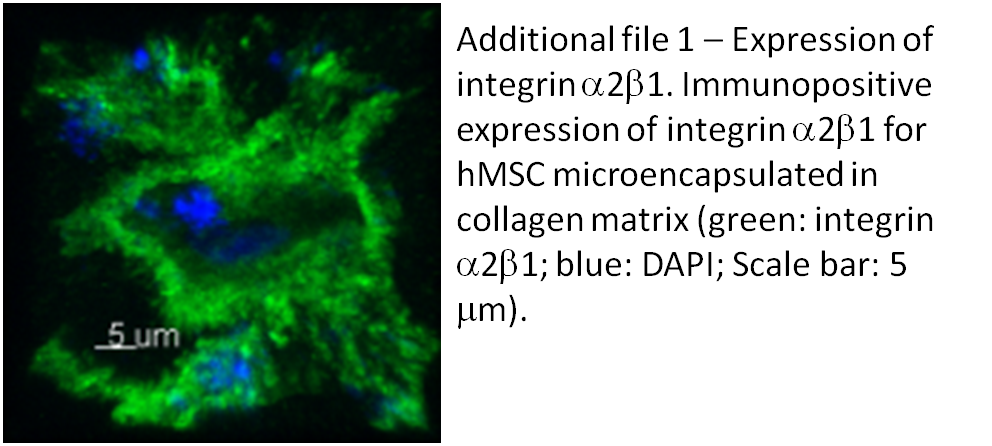

Supplement: Additional file 1: — Expression of integrin α2β1. Immunopositive expression of integrin α2β1 for hMSC microencapsulated in collagen matrix. hMSC human mesenchymal stem cell. (TIFF 452 kb) [file 13287_2015_191_MOESM1_ESM.tif]

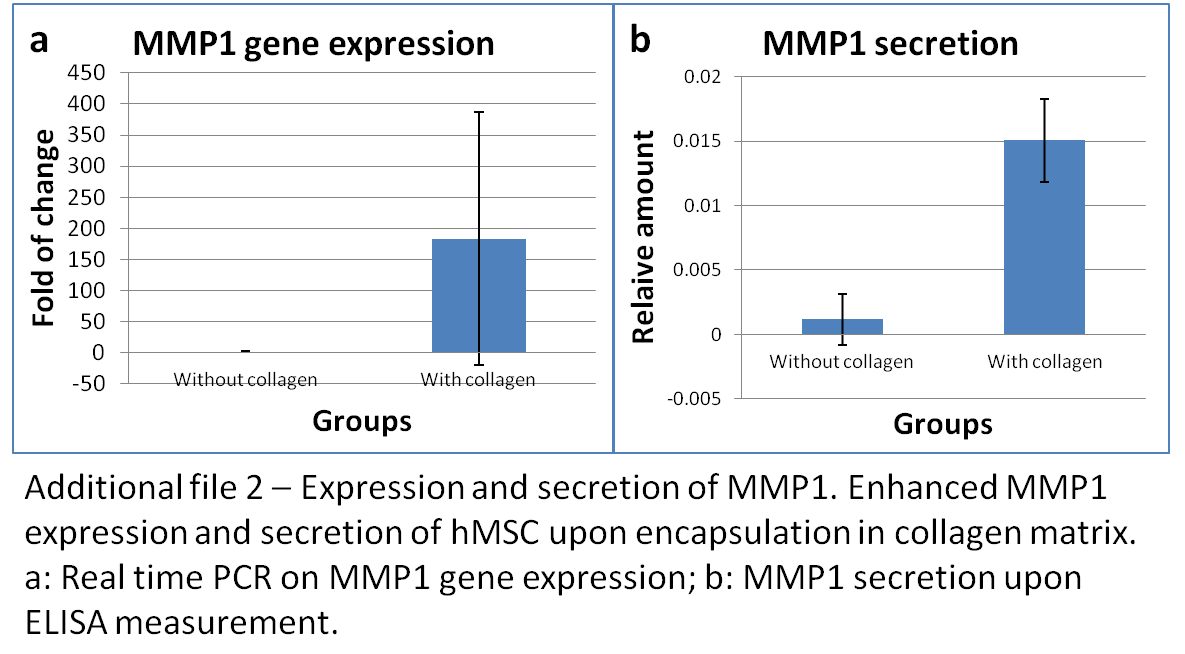

Supplement: Additional file 2: — Expression and secretion of MMP1. Enhanced MMP1 expression and secretion of hMSC upon encapsulation in collagen matrix. a Real-time PCR on MMP1 gene expression. b MMP1 secretion upon ELISA measurement. ELISA enzyme-linked immunosorbent assay, hMSC human mesenchymal stem cell, MMP matrix metalloproteinase, PCR polymerase chain reaction. (TIFF 121 kb) [file 13287_2015_191_MOESM2_ESM.tif]

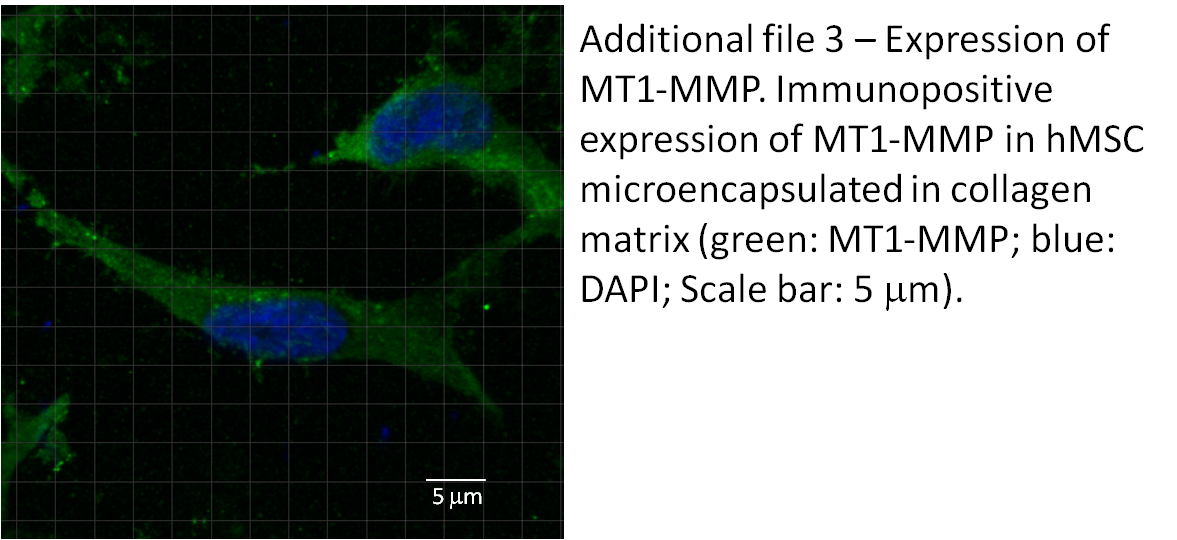

Supplement: Additional file 3: — Expression of MT1-MMP. Immunopositive expression of MT1-MMP in hMSC microencapsulated in collagen matrix. hMSC human mesenchymal stem cell, MMP matrix metalloproteinase. (TIFF 496 kb) [file 13287_2015_191_MOESM3_ESM.tif]
